# Synchrotron-based radioscopy employing spatio-temporal micro-resolution for studying fast phenomena in liquid metal foams

**DOI:** 10.1107/S0909049509001939

**Published:** 2009-03-18

**Authors:** A. Rack, F. García-Moreno, T. Baumbach, J. Banhart

**Affiliations:** a European Synchrotron Radiation Facility, 38043 Grenoble Cedex, France; b Forschungszentrum Karlsruhe – ANKA, 76021 Karlsruhe, Germany; c Technische Universität Berlin, 10623 Berlin, Germany; d Helmholtz Centre Berlin (Hahn-Meitner-Institut), 14109 Berlin, Germany

**Keywords:** X-ray radioscopy, metal foams, coalescence, CMOS, micro-radiography, liquid films, metallic thin films, viscosity, fracture, aluminium alloys, bubbles

## Abstract

Investigations of pore coalescence and individual cell wall collapse in an expanding liquid metal foam by means of X-ray radioscopy with spatio-temporal micro-resolution are reported. By using white synchrotron radiation for imaging, the rupture of a film and the subsequent merger of two neighbouring bubbles could be recorded with a time sampling rate of 40000 frames s^−1^ (25 µs exposure time) and a spatial sampling rate of 20 µm. The rupture time of a cell wall was found to be in the range of 300 µs. This value is in agreement with theoretical considerations which assume an inertia-dominated rupture time of cell walls in liquid metal foams.

## Abstract 2

High-speed synchrotron-based radioscopy is applied to study a coalescence event (which lasts ∼2 ms) *in situ* in a liquid metal foam.

## 1. Introduction

With his famous high-speed movies, Lucien Bull impressively showed the outstanding scientific value of time-resolved imaging (Bull, 1928 ▶). By using hard X-rays instead of visible light, the application fields of high-speed imaging can be extended to make visible internal structures of opaque objects as they change with time. While even fast periodic movements can be imaged by weak radiation sources *via* stroboscopic techniques (see *e.g.* Kardjilov *et al.*, 2005 ▶), the actual challenge lies in aperiodic movements that require a high density of photons for imaging. Modern third-generation synchrotron light sources with their insertion devices deliver an X-ray photon flux density high enough to perform radiography with micro-resolution in space and time (Di Michiel *et al.*, 2005 ▶; Uesugi *et al.*, 2006 ▶), currently only limited by the available detectors and the time structure of the pulsed synchrotron radiation. Imaging of triggered events using a single light pulse from an undulator source has already been reported (Wang *et al.*, 2008 ▶).

In this paper we introduce high-speed X-ray image sequences (‘movies’) captured with an image repetition rate of 40000 frames s^−1^ (FPS), which equals 25 µs exposure time. The movies image continuously the temporal evolution within a foaming metal over a time interval of several seconds. First applications of micro-radiography with a moderate acquisition speed of around 1 FPS to study *in situ* metal foaming showed the enormous potential of the method as well as a demand for higher acquisition rates (Banhart *et al.*, 2001 ▶). To measure the rupture time of a cell wall and to access the properties of the constitutive liquid metal in a single film would allow for a better understanding of the stabilization mechanisms. A recent experiment gave indication that highest acquisition rates above 10000 FPS are required for that (García-Moreno *et al.*, 2008 ▶).

## 2. Experiments

Experiments with white synchrotron radiation were carried out at the high-flux beamline ID15a of the European Synchrotron Radiation Facility (ESRF), France (Di Michiel *et al.*, 2005 ▶). The X-ray pixel detector used is based on the indirect detection concept as introduced for live topography (Hartmann *et al.*, 1975 ▶): a scintillator screen converts X-ray photons into visible light. The resulting luminescence image is optically coupled to a digital camera. In our case, the optical system is an ESRF in-house development allowing for a 1:1 projection of the screen onto the camera (Koch, 1994 ▶). As scintillating material, commercially available bulk LuAG:Ce (200 µm thick) was chosen which is known to be suited for fast synchrotron-based imaging involving high heat loads (Di Michiel *et al.*, 2005 ▶; Touš *et al.*, 2008 ▶; Banhart, 2008 ▶). The white beam of the ID15a insertion device was filtered with 25 mm of silicon, leading to an X-ray photon flux density in the range of 10^15^ photons s^−1^ mm^−2^ (ESRF, 2009 ▶). For high-speed data acquisition the novel CMOS camera Photron Fastcam SA-1 was applied (Inoue *et al.*, 2005 ▶). The CMOS chip with 1024 × 1024 pixels (each 20 µm in size) has a peak quantum efficiency of 42% at 640 nm and a ten-bit dynamic range (800:1). One signal unit (ADU) corresponds to a charge of 5.5 electrons in the corresponding potential well of the chip. The camera can acquire 5400 FPS in full-frame mode and up to 675000 FPS when using a region of interest (ROI). The minimal shutter time is 2 µs; the shutter can be triggered with a time resolution of 100 ns. The images as read out are stored in a 32 Gbyte onboard memory which defines the maximum recording length. The movies we show are taken with a ROI to reach 40000 FPS (25 µs exposure time) and an effective pixel size of 20 µm (leading to a spatial resolution *R* > 40 µm).

Aluminium-based foamable compacts were made following the powder metallurgical route, *i.e.* by mixing the elemental metal powders with TiH_2_ acting as blowing agent and pressing these. The precursors were foamed inside a furnace pressurized with argon gas. It consisted mainly of an AlMg1 tube (40 mm diameter and 0.5 mm wall thickness) with a ceramic heating plate inside. The furnace has already been described in more detail previously (García-Moreno *et al.*, 2005 ▶). The coalescence rate during foaming under normal conditions is usually in the range of some events per second in a sample of the size used. To accelerate expansion during the short available time window (a few seconds) and to provide more observable coalescence events, we depressurized the furnace during expansion. Owing to fast expansion of the foam during pressure release, a large number of coalescence events could be recorded.

In summary, the foaming procedure comprised three steps: (i) heating of the precursor under 5 bar pressure, (ii) melting of the precursor and nucleating pores still under pressure, and (iii) fast pressure release from 5 bar to 1 bar, thereby triggering fast foam expansion.

## 3. Results and discussion

**Figure 1:**
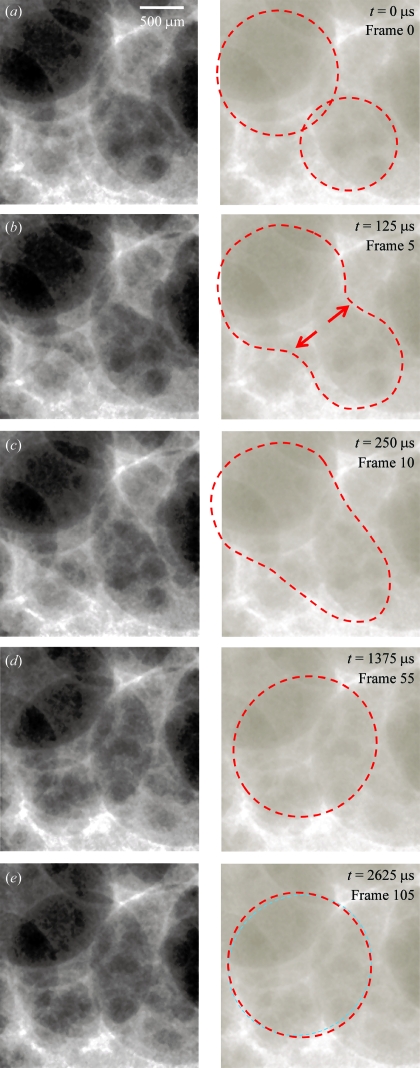
Left column: selected images of a series of 184000 showing film rupture in (*a*) to (*c*) and bubble oscillation in (*d*) and (*e*). Right column: features discussed in the text.

Fig. 1 ▶ shows a sequence of images, each of which are five or more frames apart, corresponding to 125 µs or more. To demonstrate the features that can be seen much more clearly in the moving image,^1^ the right-hand column shows the silhouette of two bubbles in the foam that merge to one larger bubble in various stages. (i) The film separating the two bubble is still intact, see Fig. 1(*a*) ▶. (ii) The film ruptures and is absorbed into the films roughly perpendicular to it, see arrows in Fig. 1(*b*) ▶. (iii) The merging dumbbell-shaped twin bubble flattens during this absorption (Fig. 1*c* ▶). The films into which the obsolete film has been sucked are flat 12 ± 3 frames (300 ± 75 µs) after the initiation of rupture [this has been termed ‘rupture time’ previously by García-Moreno *et al.* (2008 ▶)]. (iv) The two bubbles move together and form a near-spherical unique bubble after another 900 ± 50 µs. (v) The bubble continues to oscillate slowly for some time (Figs. 1*d* and 1*e* ▶).

The rupture time measured, 300 µs, can be compared with the larger value of 600 µs obtained by García-Moreno *et al.* (2008 ▶). That value corresponded to a bubble with 3 mm diameter, and a lower value is expected here since the rupture time is proportional to the diameter within the simple model presented by García-Moreno *et al.* (2008 ▶). Hence, as the diameter of the two bubbles before merger is ∼1.4mm and ∼1.1mm, and that of the merged bubble is ∼1.6mm, the rupture time measured appears to be in the range expected. This finding underlines that the rupture of a single cell wall in a liquid metal foam is dominated by inertia. The oxide particles present in the liquid metal do not prevent the moving metal from being very fluid although they form a network penetrating the entire film (Dudka *et al.*, 2008 ▶). One possible explanation is that the network is disrupted when a film ruptures, during which the apparent melt viscosity sharply drops (García-Moreno *et al.*, 2008 ▶). This also implies that the viscosity of the melt is not what makes a melt foamable as is often stated (see, for example, Song *et al.*, 2000 ▶).

In conclusion, we could measure a fast phenomenon in liquid metal foam *via* synchrotron-based radioscopy with spatio-temporal micro-resolution. The technique will allow for the investigation of film rupture in dependence of, for example, alloy composition, content of solid particles, temperature or pore radius. The current experiments only used a fraction of the photon flux density available as the high heat load of the source on the scintillating screen otherwise would irreversibly damage it. Thus, with the ongoing detector development, frame rates above 100000 FPS will soon be feasible.

## Supplementary Material

Bubbles in foam. DOI: 10.1107/S0909049509001939/kv5057sup1.avi

## Footnotes

1 Supplementary data for this paper are available from the IUCr electronic archives (Reference: KV5057). Services for accessing these data are described at the back of the journal.

## References

[bb1] Banhart, J. (2008). *Advanced Tomographic Methods in Materials Research and Engineering.* Oxford University Press.

[bb2] Banhart, J., Stanzick, H., Helfen, L. & Baumbach, T. (2001). *Appl. Phys. Lett.* **78**, 1152–1154.

[bb3] Bull, L. (1928). *La Cinématographie.* Paris: Armand Collin.

[bb4] Di Michiel, M., Merino, J. M., Fernandez-Carreiras, D., Buslaps, T., Honkimäki, V., Falus, P., Martin, T. & Svensson, O. (2005). *Rev. Sci. Instrum.* **76**, 043702.

[bb5] Dudka, A., García-Moreno, F., Wanderka, N. & Banhart, J. (2008). *Acta Mater.* **56**, 3990–4001.

[bb6] ESRF (2009). *ID15 High Energy Diffraction and Scattering Beamlines*, http://www.esrf.Fr/UsersAndScience/Experiments/MaterialsScience/ID15/

[bb7] García-Moreno, F., Babcsán, N. & Banhart, J. (2005). *Colloids Surf. A*, **263**, 290–294.

[bb8] García-Moreno, F., Rack, A., Helfen, L., Baumbach, T., Zabler, S., Babcsán, N., Banhart, J., Martin, T., Ponchut, C. & Di Michiel, M. (2008). *Appl. Phys. Lett.* **92**, 134104.

[bb9] Hartmann, W., Markewitz, G., Rettenmaier, U. & Queisser, H. J. (1975). *Appl. Phys. Lett.* **27**, 308–309.

[bb10] Inoue, T., Takeuchi, S. & Kawahito, S. (2005). *Proc. SPIE*, **5580**, 293–300.

[bb11] Kardjilov, N., Hilger, A., Manke, I., Strobl, M., Treimer, W. & Banhart, J. (2005). *Nucl. Instrum. Methods Phys. Res. A*, **542**, 16–21.

[bb12] Koch, A. (1994). *Nucl. Instrum. Methods Phys. Res. A*, **348**, 654–658.

[bb13] Song, Z.-L., Ma, L.-Q., Wu, Z.-J. & He, D.-P. (2000). *J. Mater. Sci.* **35**, 15–20.

[bb14] Touš, J., Horváth, M., Pína, L., Blažek, K. & Sopko, B. (2008). *Nucl. Instrum. Methods Phys. Res. A*, **591**, 264–267.

[bb15] Uesugi, K., Sera, T. & Yagi, N. (2006). *J. Synchrotron Rad.* **13**, 403–407. 10.1107/S0909049506023466 16924137

[bb16] Wang, Y., Liu, X., Im, K.-S., Lee, W.-K., Wang, J., Fezzaa, K., Hung, D. L. S. & Winkelman, J. R. (2008). *Nat. Phys.* **4**, 305–309.

